# Processing of metaphors in transcortical motor aphasia

**DOI:** 10.1590/S1980-57642009DN20400019

**Published:** 2008

**Authors:** Renata Mancopes, Fernanda Schultz

**Affiliations:** 1 MsC, PhD. Hearing and Speech Clinic Therapist, Language Specialist, Master in letters, Linguistics PhD, Professor of Hearing and Speech Therapy, Universidade do Vale do Itajaí (UNIVALI).; 2 Graduate student of Hearing and Speech Therapy major. Grant holder of the Research of the Article 170 of the Government of the State of Santa Catarina.

**Keywords:** aphasia, metaphor, hearing and speech therapy

## Abstract

Great emphasis has been placed on the right hemisphere, due to its possible selective contribution, in the processing of metaphorical statements.

**Objectives:**

To describe the processing of metaphors in the case of a patient with transcortical motor aphasia, using specific tests for patients with encephalic injuries of the right hemisphere, and to contribute to the discussion on the inter-hemispheric relationships associated with this function.

**Methods:**

A 54 year-old man with transcortical motor aphasia was evaluated three years after a left hemisphere stroke. The tasks of comprehension of metaphors were based on the subtest Metaphor Comprehension Task of the Montreal Evaluation of Communications Scale (MEC). Two metaphor comprehension tests were applied, in 45-minute sessions with a 48 hour interval between each. Test 1 involved comprehension of the metaphors according to the options offered, and Test 2 the comprehension of metaphors measured by response time and visual field.

**Results:**

Although the right hemisphere was not affected by the stroke in this case, difficulties were observed in the processing of metaphors.

**Conclusions:**

This study suggests that the left hemisphere participates in the processing of figurative meanings. The adaptability of the brain can also re-accommodate the uninjured areas of the brain, causing the dynamic of the brain to be modified. As a result, deducing cerebral functions based on clinical data can be problematic. The value of this study is that it can contribute to clinical aspects of language rehabilitation.

## Abstract (pt)

Um grande destaque ao hemisfério direito tem sido dado pela sua possível contribuição seletiva no processamento de enunciados metafóricos.

**Objetivos:**

Descrever o processamento de metáforas em um caso de um paciente com afasia transcortical motora por meio da aplicação de testes específicos para lesados encefálicos no hemisfério direito, a fim de contribuir para discussão das relações inter-hemisféricas associadas com esta função.

**Métodos:**

Um homem de 54 anos com afasia transcortical motora foi avaliado três anos depois de um acidente vascular que afetou o hemisfério cerebral esquerdo. As tarefas de compreensão de metáfora adotadas neste estudo de caso foram baseadas no subteste “Metaphor Comprehension Task” da Escala de Avaliação da Comunicação de Montreal. Foram aplicados dois testes para compreensão de metáforas em sessões de 45 minutos com intervalo de 48 horas entre uma sessão e outra. O Teste 1 envolveu a compreensão de metáforas segundo opções oferecidas e o Teste 2 a compreensão de metáforas medidas por tempo de resposta e campo visual.

**Resultados:**

Embora, o hemisfério direito nesse caso esteja preservado, observou-se dificuldades para o processamento de metáforas.

**Conclusões:**

Este estudo sugere que o hemisfério esquerdo participa no processamento de sentidos figurados. A adaptabilidade do cérebro pode também reacomodar as áreas ilesas do cérebro fazendo com que a dinâmica cerebral seja modificada e desse modo, deduzir funções cerebrais a partir de dados clínicos pode ser problemático. Acredita-se que este estudo possa contribuir para aspectos clínicos na reabilitação da linguagem.

Aphasia is an alteration in language processes due to neurological damage. The language functions become disorganized or restricted according to the level of central nervous system (CNS) injury, consequently limiting social and family interactions. The injuries that cause this pathology are characterized as located in the dominant cerebral hemisphere, which is generally the left for language, where some correlations have been found between aphasic syndromes and injuries in specific cerebral areas.^1^

The principle that underlies the development of the concept of aphasia was based on the motor and sensory system functions involved in the language acts of perception, interpretation and execution of signs to be controlled by at least two independently organized cerebral systems.^2^

The impaired areas that compromise language functions extend from the Broca area to the Wernicke area, angular circumvolution and white matter. Capsular and putaminal injuries can also significantly compromise communication.^3^ Etiology comprises injuries caused by cranium-encephalic traumas, tumors; infection processes of the CNS and most frequently, cerebral vascular accidents.^4^

Patients with aphasia are unable to properly convert the sequence of mental representations that constitute thinking into language signs, with similar difficulties for grammatical organization. These specific symptoms impact language function to varying degrees and are caused by dysfunction in specific cerebral regions.^4^ The individual cannot make use of the propositional language that is acquired by means of elaboration of abstract acts in relation to the situations of everyday communication.^5^

Aphasias can be classified by language behavior, into Boca aphasia, Wernicke aphasia, conduction aphasia, transcortical motor aphasia, transcortical sensorial aphasia, amnesic or anomic aphasia, and global aphasia.^1,5-7^

The language alterations hamper communication rendering the individual unable to carry out professional activities, depending on his/her occupation, often making the simplest family contact impossible. The communication limitation creates a high level of anxiety for the aphasics and their family. As a result of this picture, the individual can lose motivation to interact particular in severe deficit cases.^8^ In this context, comprehension and processing of metaphors are key factors for effective interactive communication.

These diagnoses can be carried out by the examiner using standardized tests or otherwise. An evaluation of the language to establish the aphasic topology covers as principal aspects: oral language comprehension and expression, appointment, repetition, oral reading, written language comprehension and expression, automatic language and speech fluency.^3,8^

Transcortical motor aphasia is a kind of aphasia which stems from lesions to the pre-frontal convexity (expansive injuries particularly) which can characterize certain patient disturbances in language behavior such as: anomy, which can persist independently for a considerable period of time, as well as problems with motor spontaneity.^9^ In transcortical motor aphasia although comprehension is preserved, speech is not fluent because there is impeded initiative, latency in answers and reduced medium phrase length with simplification of grammatical forms. The repetition is mainly of fragments of the individual’s utterances in situations of dialogue and requests.^10^ Transcortical motor aphasia, therefore, can be considered part of the aphasic category that does not present comprehension deficits.

With regard to language processing, some studies suggest that the right cerebral hemisphere is predominant for processing aspects such as glyph readings, assessment of metaphors and other semantic functions, rejecting the notion that this hemisphere acts only as support for the left hemisphere in language processings.^11^ Other research suggests that both hemispheres process language information as a network in parallel, even though the left hemisphere dominates for direct language tasks.^12-14^ Moreover, at all processing levels, each hemisphere computes the *input* independently and contributes toward understanding separately.^11^

We can see a relation between the speech cognitive processing and its neural base, and the activation that occurs in the right hemisphere of wide word semantic fields most remotely associated is called *coarse semantic coding hypothesis*.^13^ Metaphor processing would thus be part of the global function of this hemisphere, since a certain distance from concrete word meanings is required in order to grasp metaphors. The possible inferences of meaning activated in this hemisphere by the relationship between the topic and the metaphor vehicle would furnish the interlocutor with the proposed scope of meaning of the speaker.^11^

In another view, metaphors are understood as categorical assertions whereby the dual reference theory postulates that a lexical item such as *time bomb*, for instance, when used metaphorically designates two different referential categories: the category of bombs that explode with a programmed timer (literal sense) and the category of things that are potentially explosive and destructive over time (metaphoric sense).^15^ In this way, when the interlocutor hears or reads the sentence “Cigarettes are time-bombs”, he performs metaphor processing activating the vehicle’s non-literal meaning. From the interaction between the topic and relevant vehicle dimensions, metaphor interpretation is derived. In this theory, the metaphor is considered as a category of inclusion.^11^

Complementing the theory above, it is believed that the interaction between the topic and the vehicle is also governed by combinatory types of lexical nature. Therefore, in the metaphor “my computer is temperamental”, the topic is an artifact and the vehicle is a human character. In this kind of combination, the relevant dimension for the topic is the way in which the artifact is working. Identification of the nature of the combinations between topics and vehicles in the metaphor expressions leads to specific interpretations for each type. This allows generalizing of several metaphor combinations between different words and helps explain why only certain topic-relevant dimensions are selected in the processing of a metaphor while others are discarded.^16^

The aim of this paper was to describe metaphor processing in a transcortical motor aphasic patient by applying specific tests for cerebral injuries in the right hemisphere^11^ and in turn to contribute to the discussion on inter-hemispheric relationships for this function.

## Methods

A 54 year-old man, an electrical engineer, who had an ischemic encephalic vascular accident on November 17, 2003, was tested for the processing of metaphors 3 years after the stroke.

The language assessment followed the evaluation protocol of the UNIVALI Hearing and Speech Therapy major, evaluating both language comprehension and expression. With regard to comprehension, it was observed that the patient was able to answer when called, to understand situation orders with three or more actions, requests or commentaries, to identify verbal absurdities and to understand short histories following the therapist’s indications. However, he presented difficulties in understanding metaphors in situations with figurative language. But in relation to expression, as for the language aspects the phonetic and phonologic levels, as well as the semantic and lexical morphosyntactic were observed. No changes at phonetic and phonologic levels were observed. In contrast, at semantic and lexical levels the presence of semantic paraphasias was identified in naming tasks, in which the patient indicated the function of the objects instead of their names. However, as for the vocabulary, although anomies, the lexical items denoted a formal language level linked to the patient’s level of schooling. Concerning morphosyntactic aspects there was a predominant use of statements with three words or more, where language was not telegraphic and presented the correct use of conjunctions, gender and plural, showing occasional difficulties in the use of time and verbal conjugations.

For discursive activities the position of the subject in the lecture and narrative output was assessed. The use of personal and possessive pronouns as well as proper use of deixis was observed. In the dialogic activity there were alternating turns and discursive dependence on the interlocutor. In the construction of the story based on visual stimulations, the patient demonstrated ease of understanding for the task in hand, presenting a predominantly descriptive discourse from the outset. The subject subsequently, with the aid of the therapist, retold the narrative once more, using the narrative operators such as “and”, “so”, and “then”. The patient talked about places and characters, reasonably linking situations and facts together. However, he also presented in his discourse relevance of one of the elements of the picture in detriment to the others, and did not add new elements different to those that had already been presented, having needed the help of the interlocutor for the narrative production. In the construction of narratives from sequential visual stimulations, he demonstrated to have easily understood the task to be done, performing the sequence organization with the help of the therapist; in this task the discourse was predominantly descriptive, linking the situation with the use of cause and temporal nexus. For the reproduction activity of a story told beforehand by the therapist, it was observed that the patient paid attention to the therapist’s speech, easily understood the task to be done and produced predominantly descriptive narrative with use of operating elements of the narrative, talking about places and characters, repeating the presented facts while keeping to the suggested subject. All the tasks were performed with the therapist’s help, during which there was the presence of anomies and semantic paraphasias, which with the help of the therapist were suppressed and/or substituted. It was observed that the fluency of the dialog was maintained despite the physiopathological symptoms present in his discourse. The discourse was hesitant and the patient often restarted his sentences to reformulate what he wanted to say.

In relation to the written language, at the time of the evaluation slow reading was noted, but with adequate comprehension. However, writing could not be evaluated because the patient was right-handed, but presented right hemiplegia and showed no willingness or availability to write with the other hand.

The CT scan revealed a hypodense lesion in the basal ganglia, internal capsule and left periventricular white matter. Magnetic resonance imaging performed a week after the stroke revealed a large hypodense area compromising the left frontal, temporal and parietal regions with extension to the striatum and also internal capsule of the same hemisphere, indicating the possibility of a large ischemic stroke in the left middle and anterior cerebral artery territories.

The diagnosis of transcortical motor aphasia was made and subsequently, the patient began to be therapeutically treated, twice a week in the clinic school of UNIVALI Hearing and speech therapy. It should be emphasized that the studied case shows characteristics closer to speech motor disturbance, given low fluency speech, preserved repetition capacity, good reading comprehension and comprehension for daily activities and calculation.

All sessions were recorded and transcribed for further analysis on language functioning and case evolution. Due to clinically observed discrepancies between the general comprehension capacities of the language, which always remained adequate, and the perceived difficulty for comprehension of figurative meaning, it was decided to apply tests for metaphor processing evaluation and compare them to the results from a study^1^ which investigated subjects with right cerebral hemisphere injured brains.

The tasks of comprehension of metaphors used in this case study were based on previous research^11^ which was also used the subtest *Metaphor Comprehension Task of Montreal Evaluation of Communications Scale *(MEC).^17^ At the time this study was performed, the Brazilian version of the MEC battery^18^ had not yet been released. The test in Portuguese followed robust criteria so it is possible to establish equivalence between the stimuli in Portuguese used in the work and the current version recently released in Brazil.

The tests were applied in two forty-five-minute sessions, with about 48 hours of interval between the sessions. The tests evaluated the accomplishment of two tasks that involved fourteen metaphoric statements with a relative degree of conventionality formed by four or five words. All sentences followed the criteria of combination of semantic types that appear in the topic position and vehicle.^11^ The instruments of research were composed of two tests, namely:

- Test 1: metaphor comprehension through given options.
- Test 2: metaphor comprehension, time to answer and visual field.

In test 1 of metaphor comprehension through given options, the patient was presented a card with a metaphoric sentence and after the reading, presented a new card with three options for answers, as possibilities of interpretation of the metaphor. The options for each metaphoric sentence were composed of three explanatory sentences which attributed each a different meaning for the tested metaphor. The patient had to choose the alternative that would best explain the metaphor in the sentence (Table 1).

**Table 1:** Presentation of the patient's answers to test 1.

Metaphors		Appropriate answer		Patient's answer	
1.	This university is a hospice.	( )	There are a lot of weird people.	( )	There are a lot of weird people.
2.	This woman is turbocharged.	( )	She has silicone in her breast.	( )	She gets everything she wants.
3.	Carla is a multimedia woman.	( )	She does lots of activities at the same time.	( )	She transmits information to the public through the media.
4.	My cousin is a fridge.	( )	She is reserved in her emotional relationships.	( )	She is reserved in her affective relationships.
5.	Paulo is a bulldozer.	( )	He is aggressive with the people.	( )	He cuts trees on the farm.
6.	Marta is a rocket.	( )	She is really dynamic.	( )	She is really dynamic.
7.	Pedro is a sour guy.	( )	He is always in a bad mood.	( )	He is always in a bad mood.
8.	Paulo is a deadweight.	( )	He is a boring guy.	( )	He carries clothes on trips.
9.	Ricardo is a sweet man.	( )	He is gentle.	( )	He is gentle.
10.	Maria is a robot.	( )	She does everything well.	( )	She does everything well.
11.	My house is a hotel.	( )	It's always open to receive people.	( )	It has clean sheets everyday.
12.	My city is a Disneyland.	( )	There are a lot of options for fun there.	( )	It has crowded hotels during the whole year.
13.	My computer is temperamental.	( )	It only works sometimes.	( )	It has the latest software.
14.	My work is a prison.	( )	It's tough.	( )	It's tough.

Test 2 for metaphorical comprehension was constituted in the register of time of answer and visual field. For this the same metaphors from the previous test were used, with the help of the computer as a vehicle to apply the test. The techniques of the divided visual field was used. The technique of the divided visual field is based on the fact that when a person fixes on a central point, each eye sees both visual fields, but directs the information about the right visual field to the left hemisphere and vice-versa. This way, it is possible to send visual information to one of the side of the brain, by asking to the person to fix their gaze on a particular point, while impulses are quickly presented to each mid visual field independently.^11,19^

In this test, the metaphoric sentences were presented in the center of the screen and in the sequence the beginning of an explanatory sentence was presented. The explanatory sentence was incomplete and had to be completed with graduated and not graduated meanings by the topic interaction and vehicle suggested, be it in the right visual field or in the left visual field. Between the presentation of the explanatory sentence and each of the words for completing it, a cross appeared in the center of the screen. The patient chose the metaphoric combination with its meaning pushing the corresponding button to the answer that was judged most appropriate. The focus of interest was the divided visual field, reaction time and number of correct answers. The buttons yes and no were indicated on the mouse of the computer, corresponding to the green color for YES and the red color for NO. The test configuration was thus established:

- Back color: light blue;
- Size, type and color of letters: 55, tahoma, dark blue;
- Initial delay: 3300 ms;
- Metaphor: 2500 ms;
- Start of the explanatory sentence: 2000 ms;
- Fixation sign: 1500 ms;
- Answer presentation: 1000 ms;
- Time between answers: 2000 ms;
- Time between the last answer and next metaphor: 3500 ms.

Before the test, training was performed consisting of six training-sentences already used in the previous activity, repeating the training the number of times asked by the patient (three times) until he felt ready to undergo the definitive test. The instructions for test 2 were presented by the researcher. Initially a sentence appeared in the screen for about 2500 ms and soon afterwards, the beginning of the explanation of the sentence appeared for 2000 ms, disappearing after this time. After this the words comprising the explanation appeared on the screen individually. Each word appeared randomly on the right or left side of the screen for about 1000ms. The patient had a time of 2000 ms after the word had vanished to answer YES or NO. Whenever he could not answer in time, he lost the chance and a new word appeared on the screen. Between the sentences and the words, a cross was displayed in the middle of the screen for 1500 ms.^11^ The answers obtained in the test were registered and stored on the computer.

The patient was informed about the characteristics of the study having signed the term of free and agreed consent, authorizing the publication of the data presented here.

## Results

Test 1 previously demanded that the patient marked the answer to explain the metaphor presented to him on the card. It was noted that the patient needed to read each sentence in the analysis twice, while sentences 4, 6 and 9 were read three times by the patient before indicating his answer.

The answers marked by the patient were as follows (Table 1).

It was observed that, out of all metaphorical sentences presented to the patient in test 1, he achieved 50% correct answers. The answers given in the sentences 3, 5 and 8 refer to the literal meaning of the expressions under analysis. In sentence 12, due to the need for marking one of the answers the patient said “it looks the same” and finally marked the third option.

The appropriate answers to test 2 are described in Table 2. The quality of the answers was considered “expected” when the patient answered with the appropriate answer as presented in Table 2 and considered “not expected” when he answered differently from the appropriate answer. The results obtained in the patient’s test are the ones that follow in Table 3.

**Table 2:** Presentation of the appropriated answers to test 2.

Metaphors	Explanatory phrase beginning	Answers
This university is a hospice.	There are only... people there.	weird
This woman is turbocharged.	She has	silicone.
Carla is a multimedia woman.	She does lots of… at the same time.	activities
My cousin is a fridge.	She is	reserved.
Paulo is a bulldozer.	He is	aggressive.
Marta is a rocket.	She is	dynamic.
Pedro is a sour guy.	He is	(always) in bad mood.
Paulo is a deadweight	He is	boring.
Ricardo is a sweet man.	He is	gentle.
Maria is a robot.	She is	perfectionist.
My house is a hotel.	It is	Welcoming.
My city is a Disneyland.	It is	fun.
My computer is temperamental.	It is	unstable.
My work is a prison.	It is	tough.

**Table 3:** Distribution of the answers of the metaphors.

Metaphors	Possibilities	Visual field	Time for reaction (ms)	Kind of answer	Quality of the answer
1. This university is a hospice.	Weird	Right	Didn't interact	Didn't interact	Not expected
	Medicated	Left	2364	No	Expected
	Wet	Right	1512	No	Expected
2. This woman is turbocharged.	(To be) strong	Left	2053	No	Expected
	Silicone	Right	1723	No	Not expected
	Acne	Left	1412	No	Expected
3. Carla is a multimedia woman.	Activities	Right	2454	Yes	Expected
	Races	Left	1703	No	Expected
	Reports	Right	2013	Yes	Not expected
4. My cousin is a fridge.	Freezing	Left	2043	No	Expected
	Heavy	Right	1612	No	Expected
	Reserved	Left	2704	No	Not expected
5. Paulo is a tractor.	Vehicle	Right	1672	No	Expected
	Heavy	Left	1663	No	Expected
	Aggressive	Right	2454	No	Not expected
6. Marta is a rocket.	Spacecraft	Left	2023	No	Expected
	Gossiper	Right	2154	No	Expected
	Dynamic	Left	1933	Yes	Expected
7. Pedro is a sour guy.	In bad mood	Left	1993	Yes	Expected
	Acid	Right	1542	Yes	Expected
	Ironic	Left	1402	Yes	Expected
8.Paulo is a suitcase.	Locker	Right	1803	No	Expected
	Handsome	Left	1842	No	Expected
	Boring	Right	1452	No	Not expected
9. Ricardo is a sweet guy.	Gentle	Left	1923	Yes	Expected
	Flatterer	Right	2154	No	Expected
	Handsome	Left	1963	No	Expected
10. Maria is a robot.	Perfectionist	Right	2374	Yes	Expected
	Made of metal	Left	1643	No	Expected
	Rechargeable	Right	1572	No	Expected
11. My house is a hotel.	Welcomer	Right	2975	Yes	Expected
	Clean	Left	2333	Yes	Not expected
	Luminous	Right	1993	No	Expected
12. My city is a Disneyland.	Tourist	Left	2794	Yes	Not expected
	Fun	Right	2483	Yes	Expected
	Dirty	Left	2043	No	Expected
13. My computer is temperamental.	Unstable	Right	2133	No	Not expected
	Salty	Left	1553	No	Expected
	Complicated	Right	2343	No	Expected
14. My work is a prision.	Badly-paid	Left	2554	Yes	Not expected
	Liberal	Right	1913	No	Expected
	Tough	Left	2143	No	Not expected

It was noted that there were some differences in the answers for some metaphors in test 2 in relation to test 1, although the patient gave some of the suggested answers correctly. The 50% success rate in the answers was repeated as in test 1. It was also observed that he maintained literal interpretations, for instance on the metaphors 3 and 11. Moreover, it is important to mention that when he answered in an unexpected way in relation to the appropriate answer, and in an expected way in relation to the other two options (that were the wrong ones), one cannot be sure that he has accessed the meaning of the metaphor in fact or of both senses marked as expected. The difficulty presented by the patient may be in rejecting or otherwise, the alternative meaning of the experimental impulse, and not strictly in the understanding of the metaphor.^20^

In metaphor 3 it was observed that although the patient was right about the metaphorical interpretation, he also marked as a possible interpretation the option that refers to the literal meaning. It is relevant that in test 1 the patient had also answered with the literal meaning as for the interpretation of this same metaphor.

As for metaphor 4, the patient did not answer in an expected way in test 2, while in test 1 he had answered appropriately. As for metaphor 8 it was observed that the patient did not answer in an expected way in test 2 either, and in test 1 he had also maintained the literal interpretation.

In the interpretation of metaphors 11 and 12, although the patient had accessed the metaphorical sense answering in an expected way to these two interpretations, it was also observed that possible meanings were accessed in the literal relationship of the terms. In general, a difficulty was seen in the processing of metaphors on the part of the patient, who in 50% of the cases accessed the metaphorical interpretation of the statements, but seemed to remain bound to the literal meaning of some expressions. Although the test identified the time of the answer and visual field, these were not taken into account for this study, for they constitute subject of future studies.

## Discussion

For some time the right hemisphere was conceived inappropriately as the least important or the passive hemisphere. Over the seventy years since Broca’s discoveries regarding the left hemisphere, the role of the right hemisphere has remained unknown. It seemed that the right hemisphere was capable of tolerating greater injuries without presenting evident damage. Small injuries in certain areas of the left hemisphere drastically affected the abilities of speech, whereas similar injuries in the right hemisphere did not seem to cause any serious dysfunction. This disparity was interpreted at first as a less important role of the right hemisphere in human behavior. More recently, however, it has been proposed that this difference simply reflects the way the processes are organized in the right hemisphere, and some authors^21^ believe that specific processes are distributed in wider areas of the right half of the cerebral fabric than in the left half. And, in spite of its different role, the right hemisphere carries out a vital role in human behavior and it is already quite clear that both hemispheres contribute to the complex mental activity, although differing in their functions and organizations.

Specifically, as for the processing of metaphors, studies with normal subjects^22-24^ have revealed the direct involvement of the right hemisphere in the processing of metaphorical language. Patients with injuries in the right hemisphere have difficulties to interpret indirect speech,^25,26^ connotative semantic relationships,^27,28^ phrasal metaphor^30^ and lexical metaphorical dimension.^14,27^

In spite of their preserved linguistic abilities, clinical observations of patients with injuries in the right hemisphere show that they possess preferences for literal interpretations, rejecting, therefore, metaphorical implications. As metaphoric language represents a linguistic resource highly used in daily speech at formal or informal levels, not being able to process it interferes in the understanding of discursive occurrences, leading to isolation from society. This aspect turns out to be of extreme relevance when associated to the therapeutic objectives of language rehabilitation.^11^

The first investigation studies subjects with injured brains for metaphorical understanding^29^ using the paradigm of the matching between sentences and engravings. They found dissociation among the pictorial and verbal conditions in the patients with injuries to the right hemisphere, who interpreted the metaphors in a literal way when it involved the pictorial task, but who interpreted them correctly when they were requested to explain them verbally. The applied tests required patients with injury in the right hemisphere and the control group, to perform matching of sentences, such as *“He had a heavy heart”*, to an engraving, which was represented by an illustration of the literal meaning of the sentence (a man lifting a heavy heart), one of metaphorical meaning (a man crying), and for different aspects of the literal meaning (an illustration with an enormous weight, and illustration of a heart). In relation to the normal group, both patients with injuries in the right hemisphere and left hemisphere presented problems, but, when compared to one another, the patients with injuries in the right hemisphere presented a larger number of mistakes for each choice, more frequently opting for the literal engraving.^11^

A similar task was applied in aphasic patients with injuries to the left hemisphere who presented better performance than the patients with injuries to the right hemisphere in the crossing of words, such as *wealth*, with the connotative pictorial representation of an arrow pointing up or down.^28^ In comparison of abilities related to language between patient with injuries in the right and left hemispheres, good performance was found in both groups on the understanding of isolated words; the patients with injuries to the right hemisphere understood new sentences better, while individuals with injuries to the left hemisphere understood familiar idiomatic sentences better.^30^

Considering that patients with injuries in the left hemisphere tend to have more perceptive language problems and that they have better performance than the patients with injuries to the right hemisphere on the tasks of understanding of the figurative language, a special role can be attributed to the right hemisphere in the understanding of figurative language. A possible reason for the superiority of the left hemisphere with injuries in the accomplishment of the tasks outlined; however, it is that they involved the matching of sentences with engravings. While engravings possibly served as additional information to the patients with left hemisphere with injuries, they presented difficulties for the right hemisphere subjects, for most of them had visuospatial deficit. It is known that patients with injury in the right hemisphere would normally present worse results in non-verbal tests involving manipulation of geometric illustrations and tasks including forms.^21^ Patients with injury in the right hemisphere, however, also showed problems with metaphorical meanings in purely verbal paradigms.

Studies using the paradigm of triads of words, for instance, *cold – hateful – warm*, requested the participants to match words that had the same meaning or that best went together. Semantic relationships among the words were based on the denotative relationships (former: antonyms cold and warm), connotative (*cold* and *foolish*), metaphorical (*cold – hateful*) or not related (*cold* and *wise*). The performance of patients with right hemisphere injuries was normal for the use of association of antonyms, but below normal for the metaphorical equivalence.^27^ The opposite occurred in patients with left hemisphere injuries. A possible explanation for this impairment could be the difficulty in recognizing and attributing less frequent meanings of ambiguous words, rather than the strict recognition of metaphorical meanings.^11^

The test of the metaphorical and non-metaphorical adjectives, although ambiguous, was also accomplished by means of two tests in a triad task and a dual task. In the first, participants had to identify which presented the most similar meaning and, in the second, the plausibility in relation to the two words. In relation to the normal group, both groups with injuries presented difficulty in the accomplishment of the task.^31^ Such studies indicate that injuries in the left hemisphere provoke a decrease of sensitivity for the literal aspect of meaning and increase the dependence of connotative and metaphorical meaning. Damage in the right hemisphere, on the contrary, seems to increase dependence of the literal aspect of the word with loss of sensitivity for connotative and metaphorical aspects. However, the results presented here do not seem to corroborate with this notion, since the case analyzed performed exactly in the opposite way, suggesting evidence for dependence for literal meaning.

The studies previously presented refer to the difficulty of right hemisphere brain injured subjects in the processing of the metaphors; however other results^11^ presented for the same test revealed that most of the answers of the researched group were right for the metaphorical interpretation of each sentence. Only five of the participants of the study opted to not to mark any of the alternatives, which can be evidence of difficulties in rejecting the alternative meanings. In any event, the patient-case in this study maintained a 50% rate of appropriate answers and demonstrated difficulty in processing alternative meanings even if there are no cerebral injuries in the right hemisphere, but rather in the left. This fact makes may suggest that cerebral connections are dynamic and that maybe it is not plausible to attribute the function of processing figurative senses only to the right hemisphere.

Studies conclude that people with unilateral cerebral injury maintain the right hemisphere neurologically intact, process several types of figurative language, although they present damaged structural and formal aspects of the linguistic system.^20^ The review of the research carried out by another study^32^ shows that the difference in the communicative performance between right and left hemisphere-injured patients tends to demonstrate that separate components of language are compromised after a right or left injury and in contrast to what is presented in most of the literature,^33,34^ do not confirm the average of 50% of right hemisphere injured with communicative problems. In the three studies consulted on the processing of metaphors after injury to the right hemisphere, either no significant difference was found between people with injuries in the right or left hemisphere^35,36^ or just half of the group presented difficulties.^37^

Consequently, deducing a cerebral function based on clinical data is problematic, given that the brain possesses a tendency for adjusting its operations in the best possible way after an injury; which does not mean that remaining intact areas of a injured brain operate as they would in a normal brain. It is not as if when “lacking a piece” everything else would work as before.^21^ In most cases of cerebral injuries, some function recovery occurs over time and sometimes remarkable recovery is possible. Recovery can involve changes in the intact areas and representing the contribution of brain adaptability.

Other authors criticize studies carried out with right hemisphere injured patients because they consider that in most cases there seems to be no solid evaluation for perceptual deficits characteristic of these cases, or a detailed analysis of the patients’ linguistic abilities. Besides, they emphasize that in general, the sample is small and because they use a paradigm of forced choice the results may be related to the difficulty presented by the damaged right hemisphere in rejecting the alternative meaning of the experimental impulse or not, and not strictly to metaphorical understanding.^20^

The presented studies and the case study proposed in this work revealed divergences in the results found by researchers which call in to question the specificity of the right hemisphere for the understanding of figurative language. It was noted in these studies that not all of the participants with injuries in the right hemisphere showed such deficits and that, in the outlined case above there were difficulties for metaphorical understanding, despite being a case of left cerebral injury. In fact, the conducting of more research seems needed which could enlarge the scope of the subject, since the studied case suggests more complex and dynamic relationships between the cerebral hemispheres for understanding of metaphors. Specifically in this case, as regards to transcortical motor aphasia, the patient presented deficit of 50% for both proposed tests in the metaphorical understanding and evidenced linguistic dependence to the literal meaning for processing of figurative sense. Besides, it seems problematic to deduce cerebral functions based in clinical data since the adaptability of the brain can also readapt in the intact areas thus modifying the cerebral dynamics. Therefore, in spite of being a fundamental and useful characteristic, plasticity can, as regards to rehabilitation, complicate researchers’ efforts to deduce cerebral functions based on clinical data.
